# Conceptualising the experience of having TB: a global qualitative study

**DOI:** 10.5588/ijtldopen.25.0112

**Published:** 2026-01-09

**Authors:** D. Oberdhan, M. Hill, A. Gillman, N. McGale, J. Dandy, T. Abdullaev, M. Dara

**Affiliations:** 1 Otsuka Pharmaceutical Development and Commercialization, Inc., Rockville, MD, USA;; 2 Faculty of Health Sciences and Wellbeing, University of Sunderland, Sunderland, UK;; 3 Modus Outcomes, A THREAD Company, London, UK;; 4 Real Chemistry, Philadelphia, PA, USA;; 5 TBpeople Global, London, UK;; 6 Otsuka Novel Products GmbH, Munich, Germany.

**Keywords:** tuberculosis, quality of life, disease impact, linguistic analysis, social media, social determinants of health

## Abstract

**BACKGROUND:**

TB and aspects of its treatment are known to impair health-related quality of life, but data are lacking on how pulmonary TB is experienced from the standpoint of affected individuals.

**METHODS:**

We conducted a review of published literature and online content with the goal of conceptualising the personal experience of TB. Using social media sources drawn from the years 2020–2022, concepts from the literature review were updated with the perspectives of people with TB and caregivers from multiple countries across different World Bank income categories.

**RESULTS:**

The literature review identified 110 published articles and 91 stories of people with TB, enabling the generation of a conceptual model representing symptoms, disease impacts, and diagnostic and treatment challenges. Concepts of interest varied by country income level. The social media analysis captured 657 posts across 15 countries; the impacts described included physical (48%), emotional (28%), economic (18%), social (13%), caregiver (10%), and cultural (3%) impacts. Diagnosis was frequently associated with delays, access challenges, and confusion with COVID-19.

**CONCLUSION:**

People affected by TB worldwide report impacts on multiple life dimensions, but the types of impact vary by geography, income, and culture. Additional research sensitive to local experience is needed.

Active TB disease constitutes a major global health burden, with the most recent WHO TB Global Report indicating that in 2023, 10.8 million individuals became ill with TB and 1.25 million died from the disease.^1^ The most important treatment goals in active TB are preventing fatality and breaking the transmission chain. However, affected people also experience potentially severe reductions in health-related quality of life resulting from symptoms (e.g., cough, fever, and fatigue), psychological distress, impairment in daily activities, and the difficulties and economic costs of treatment.^2^ Stigmatisation in the community and discrimination pose additional challenges to affected individuals.^1^ Furthermore, newer treatment regimens for drug-resistant types of TB are effective yet associated with toxicity and adverse effects.^3^ Even after treatment, people may manifest post-TB sequelae that arise from the disease itself or its treatment, including chronic respiratory impairment, neurologic disorders, or drug-induced liver injury.^4^ Many of the hardships associated with TB are not limited to people with active disease, given that those diagnosed with latent TB may feel anxiety about developing active disease or potentially have to start antimicrobial therapy to eliminate the infection or decrease the risk of developing active disease.^5–7^

Despite the implications of TB for health-related quality of life, data on the experience of having TB and the use of validated, disease-specific assessment instruments for such research are lacking.^2,5^ According to global agencies, understanding the experience of TB is critical to the goal of ending the epidemic.^8,9^ Awareness of the challenges faced by people with TB and their families is needed to inform health policy, embed the needs of affected individuals in the development of new treatments (for example, in clinical trial design), and promote adherence to therapy.^9–12^ Methodological literature on outcomes research recognises the importance of conducting qualitative investigation early in drug development to ensure that the voices of people affected by TB shape the conceptual model and terminology used.^13,14^ National and international agencies have issued guidance on incorporating these perspectives into the evaluation of new treatments.^15–17^

The purpose of this study was to conceptualise the experience of people with TB in order to facilitate the inclusion of assessment measures that are relevant to the concerns of affected persons in future TB clinical trials. Since TB and its treatment are experienced differently worldwide, the study drew from multiple sources, including published literature and online testimony, to capture voices from across a broad array of global geographies and income levels.

## METHODS

### Literature review

A PubMed search was conducted for qualitative data on the experience of having TB as published within a 10-year period (2012–2022). Terms searched at the title/abstract level were: tuberculosis; mono-resistance TB; poly-resistant TB; rifampicin resistance TB; multidrug resistant TB; extensively drug resistant TB; drug resistant TB; drug susceptible TB AND qualitative; interview; focus group; conceptual model; conceptual framework. Articles were selected in a two-stage process in which the title and abstract were screened first and the full-text article was screened second. At each stage, articles were excluded in the following order: 1) sample did not include people with a current or previous TB diagnosis; 2) sample did not include pulmonary TB (exclude if extra-pulmonary TB); 3) not qualitative research reporting results qualitatively; 4) articles clearly out of scope and not reporting data on the experiences of people with a past or current diagnosis of TB; 5) systematic reviews or meta-analyses; and 6) not available in English.

The search for published articles was supplemented with a review of websites from medical associations and from community advocacy organisations for people affected by TB, to obtain additional data on the experience of having TB. Eligible content consisted of stories or blogs reported from the perspective of a person with pulmonary TB, with information on symptoms, impacts, and/or experiences of diagnosis and treatment (see Supplementary Data for more detail on the search strategy).

### Social media linguistic analysis

To obtain the most current data, reflective of the TB experience after the outbreak of the COVID-19 pandemic, we searched social media sources across multiple world regions. Online conversations from over 200 million sources on the Internet were searched for the period from 1 January 2020, through 30 November 2022, including blogs, forums, social networks, and other sources of public online conversation. Content in English, German, Spanish, French, Italian, and Portuguese was captured, with posts manually coded by analysts fluent in that language and experienced in analysing medical conversations in that language. Eligible posts were limited to those that discussed a personal experience with one of the following: TB symptoms, impact of having TB, difficulties associated with TB treatment, and/or coping mechanisms. Post authors could be people with TB, caregivers, or clinicians (see Supplementary Data for more detail).

### Data evaluation

Results of the literature review were collated and synthesised to conceptualise the personal experience of active pulmonary TB. Using data obtained from published articles and the web-based stories of people affected by TB, we compared symptoms and impacts across World Bank country classifications by 2022–2023 income level. The classifications were based on gross national income per capita of the previous year in US dollars: low (<$1,085), lower-middle ($1,086–$4,255), upper-middle ($4,256–$13,205), and high (>$13,205). Differences in available resources and health care systems might be expected to affect symptoms experienced and impacts of having TB. The resulting conceptual model was qualitatively compared to data obtained from the social media linguistic analysis to determine whether each methodology identified similar concepts. Additionally, data from the social media linguistic analysis were stratified by a combination of World Bank national income classification and geography to obtain insights differentiated by income and region. The group of countries of interest for the social media analysis were lower-middle income, upper-middle income, and high-income countries. In the high-income category, the United States was analysed separately from the European Union + United Kingdom due to differences in data access permissions and a larger number of posts in the United States discussing latent TB. The conceptual model development process is described in more detail in the Supplementary Data.

### Ethical statement

Ethics approval was not sought for this research, given that all of the data were obtained from publicly available sources.

## RESULTS

### Literature review

The literature review identified 110 published articles and 91 web-based stories or blogs on the experience of having TB (Supplementary Data Figure S1). The content was sourced from 48 countries (Supplementary Data Figure S2) across four categories of World Bank national per capita income classification (Table 1). Among the published articles, the distribution of publication dates was 2012–2014, 19%; 2015–2017, 24%; 2018–2020, 29%; and 2021–2022, 20%. The primary objective of the published articles was determined to be: describing TB symptoms (24%), TB life impacts (15%), TB diagnosis (8%), or TB treatment (53%). In 68% of published articles, comorbidities were not reported, whereas in 32%, comorbidities were mentioned, including HIV (22%), diabetes (7%), substance abuse (8%), and other (6%). Most of the articles reported on adults only (83%) and included both female and male participants (76%).

**Table 1.** Distribution by national income level of published articles and web-based patient stories/blogs included in the literature review.

National per capita income level^**A**^ (US dollars)	Low (<$1,085)	Lower-middle ($1,086–$4,255)	Upper-middle ($4,256–$13,205)	High (>$13,205)
Published articles (n = 110)^**B**^	14	46	44	10
Web-based patient stories/blogs (n = 91)	8	23	28	32

A World Bank 2022-2023 income classification based on gross national income per capita of the previous year.

B Two articles listed >1 country; hence, the total number is greater than 110.

The web-based stories/blogs were obtained from seven websites. The greatest number were obtained from an international, collaborative blogging project by people on treatment for multidrug-resistant TB that was featured on the Doctors Without Borders website (*n* = 46 blogs) and from personal stories by people with TB on the website for the US Centers for Disease Control and Prevention (*n* = 20 stories). The other sources were the websites for TB Alert, an international advocacy group for people with TB (*n* = 6 stories/blogs); TB Alliance, a not-for-profit alliance for developing TB treatments (*n* = 3); TB Europe Coalition, an advocacy network of civil society organisations (*n* = 6); The Truth About TB, an information service hosted by TB Alert (*n* = 6); and the World Health Organization (*n* = 4). All of the content was available in English per the search criteria, including one personal story that had been translated from Somali. The stories/blogs were from people in Armenia, Central African Republic, Eswatini, Ethiopia, Georgia, India, Iraq, Libya, Mexico, Moldova, Myanmar, Namibia, Netherlands, Papua New Guinea, Philippines, Romania, Russia, Somalia, South Africa, Sudan, Tajikistan, Ukraine, United Kingdom, United States, Uzbekistan, Vietnam, Zambia, and Zimbabwe.

**Figure 1.**
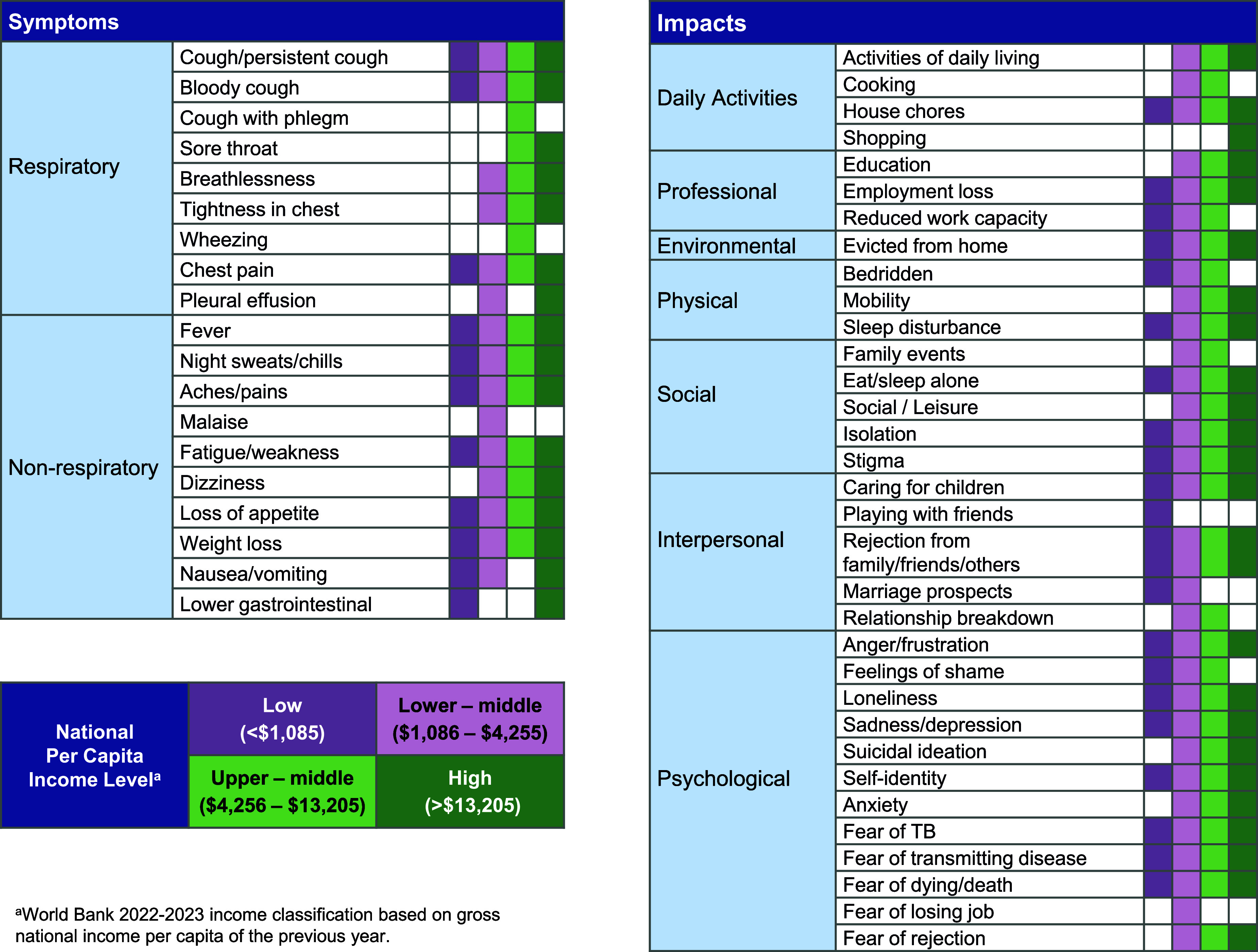
Symptoms and impacts of TB by national income level in the literature review.

Aspects of the personal TB experience from the literature review (published articles and web-based stories) were collated to develop a conceptual model that was broadly representative of the hardships of disease (symptoms, impacts) and difficulties associated with TB management (diagnosis, treatment). Regarding disease hardships, people with TB described a wide range of respiratory and non-respiratory symptoms and TB impacts across national income levels (Figure 1). Coughing and fever were reported at all income levels. Emotions of suicidal ideation and anxiety were reported in the lower-middle, upper-middle, and high-income groups but not the low-income group. Feelings of stigmatisation, isolation, and anger were present at all income levels, as were fears of professional impact (e.g., loss of employment or educational opportunity) or environmental fallout (e.g., eviction from housing).

**Figure 2.**
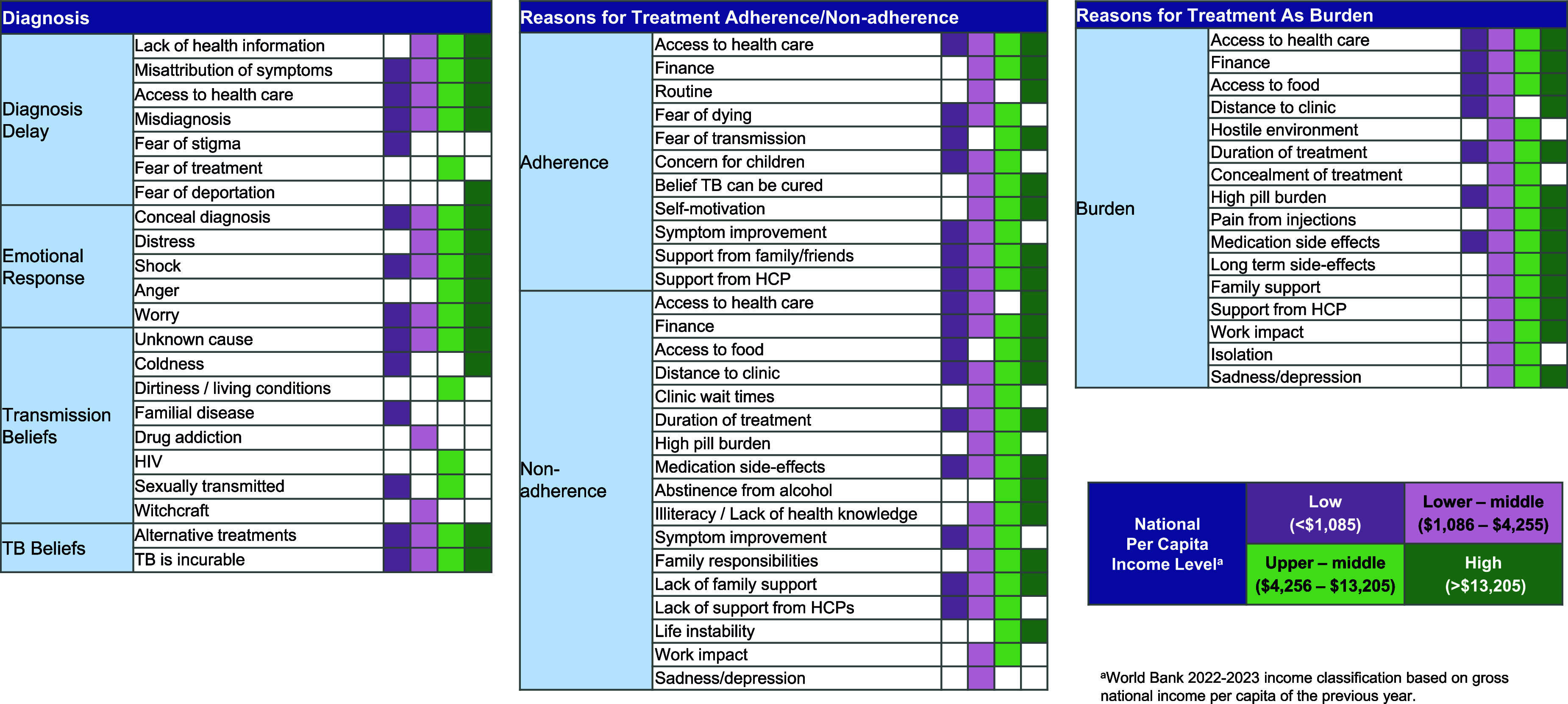
Experience of TB diagnosis and treatment by national income level in the literature review.

**Figure 3.**
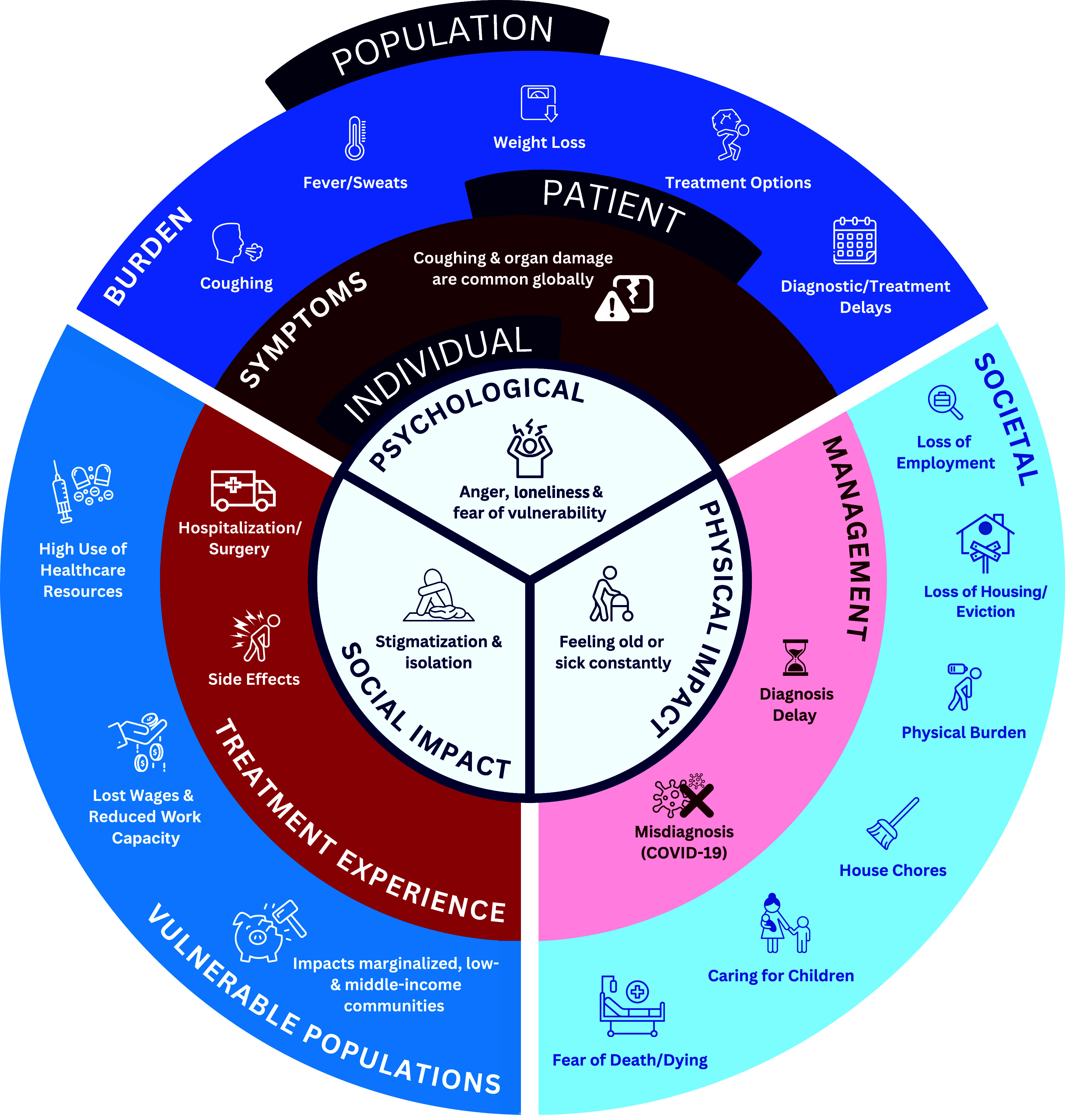
Simplified depiction of the conceptual framework developed for the experience of TB.

In the analysis of TB management, diagnostic delays, treatment delays, and challenges associated with treatment were reported across different national income categories (Figure 2). However, these experiences were also influenced by cultural factors, belief systems, and challenges related to health care access. A simplified version of the conceptual model is shown in Figure 3.

### Social media linguistic analysis

Of 4.9 million public social media posts identified from TB discussions globally, 1.4 million originated from the regions of interest and were posted in the languages captured. From this pool, a total of 657 posts met eligibility criteria for inclusion, that is, were posted by people with TB, caregivers, or clinicians and discussed TB symptoms, impacts, difficulties of treatment, and/or coping mechanisms. The area of origin for the 657 posts was a high-income country for 377 posts (United States, 213; European Union [France, Germany, Spain, Italy]/United Kingdom, 164), an upper-middle income country for 92 posts (South Africa, Brazil, Peru, Thailand), and a lower-middle income country for 188 posts (India, Nigeria, Pakistan, Philippines, Indonesia). The author was a person with TB for 72% of posts and a caregiver for 28% of posts (no clinician posts met the inclusion criteria). Among posts identifying the approximate age of the person with TB (397/657), 84% identified them as an adult at the time of TB diagnosis and/or treatment. The analysed posts discussed TB symptoms (147/657; 22%), impacts (454/657; 69%), treatment (169/657; 26%), or coping (73/657; 11%) – more than one topic per post was possible. The impact of TB was the most common topic across posts in all regions: United States (75%), European Union/United Kingdom (66%), upper-middle income countries (54%), and lower-middle income countries (72%). Approximately half of all posts mentioned a type of TB; within this group (324/657), the type was latent TB in 40%, active TB in 41%, pulmonary in 14%, extra-pulmonary in 22%, and multidrug-resistant in 4%.

The hardships of TB described in social media were consistent with the conceptual model from the literature review. Coughing was the most common symptom reported across regions/income levels (25%), followed by organ damage/abnormalities (18%; most commonly lung issues such as collapsed lung, reduced lung capacity, or scarring), fever/sweats (11%), and shortness of breath (11%) – see Supplementary Data Figure S3. Other symptoms raised included weight loss (10%), aches and pains (9%), coughing up fluid (5%), and fluid build-up (5%). A few symptoms reported in the social media linguistic analysis (e.g., women’s health issues and skin abnormalities) were not observed in the literature-based analysis. An absence of symptoms was reported more frequently in posts from the United States (24%) versus other regions (3%). Among the 454 mentions of impact, physical (48%), emotional (28%), economic (18%), social (13%), caregiver (10%), and cultural (3%) impacts were identified. The frequency of physical impacts mentioned in the social media analysis did not vary by region/income level, while differences were evident in the frequency of social and emotional impacts. Economic impacts were most commonly reported at lower national income levels.

Posts on TB management showed that certain themes, such as diagnostic and treatment delays and difficulties associated with treatment, were common across different income categories. A new theme that emerged from the social media linguistic analysis versus the literature review was that the similarity of TB and COVID-19 symptoms led people with TB to believe they had COVID-19, thereby delaying the TB diagnosis, as stated in posts from the United States, European Union/United Kingdom, and upper-middle income countries. The frequencies with which other concerns were mentioned, however, varied by national income level, including hospitalisation, medication adherence, medication side effects, and barriers to accessing medical care (Supplementary Data Figure S4). Quotes from people with TB and caregivers that are illustrative of such experiences are shown in Table 2.

**Table 2.** Commonly reported themes on TB impact and management in social media posts by national income level.

Themes	Representative quotes (country)
TB impact	
USA: People with latent TB expressed fear about developing active TB infection. People with TB also felt increased vulnerability to COVID-19.	It turns out all I had was latent TB; my lungs were clear. To keep my latent TB in check, though, they suggested an antibiotic, which was intense but worth it. (US)
	I have latent TB. Looks like a life of chest X-rays and QuantiFERON-TB Gold tests because a tuberculin skin test won’t work. (US)
EU/UK: People with TB expressed anxiety about vulnerability to COVID-19.	My mother had TB when she was a child and her lungs have been permanently damaged. It could be very dangerous if she’s infected with COVID-19. (Germany)
UMI: Fear of contracting COVID-19 was the most common impact and led people with TB to self-isolate to reduce their chances of infection.	Before the pandemic, I was diagnosed with tuberculosis. I am immunocompromised and haven’t left my house because I am too scared. (South Africa)
LMI: The financial burden of treating TB was the paramount impact. People with TB and caregivers sought financial support online from their social circle, advocacy groups, and strangers.	I am the provider of my family, but I am struggling with debt after needing an unexpected medical procedure to remove an intestinal cyst. (Philippines)

TB Management	
USA: People with TB frequently mentioned specific side effects from TB medications.	I shared my experiences last night with what someone called ‘brain fog’ on the tuberculosis medication. Just this morning, I made my coffee with hot water… no coffee. With the full coffee set-up I have going, this mistake would have had me immediately calling a neurologist under other circumstances. (US)
	These TB drugs are just plain awful. The nausea, exhaustion, dizziness, headaches … it’s the worst. (US)
EU/UK: Posts from the EU/UK were more likely than US posts to mention hospitalisation and/or surgery. To a lesser extent than US posts, UK/EU posts also discussed treatment side effects.	My mother has been in the isolation ward at the hospital for 4 weeks now. She was infected with tuberculosis at work (she works with many refugee children). (Germany)
	When I had lymph node tuberculosis in 2017, I had excruciating night sweats and neon orange pee from the medications. (France)
UMI: Hospitalisation was the most-mentioned treatment topic, particularly in Brazil.	I was taken to a hospital specializing in lung diseases, where I was diagnosed with TB. I was never a smoker or drinker, but I contracted TB because I have bronchiectasis since I was born. (Brazil)
LMI: Hospitalisation and surgery were mentioned often among people with TB in the lower-middle income region. Financial and non-financial barriers to medical care were common themes.	In January, I had Thoracic Spinal Fusion surgery to treat Bone Tuberculosis. I was operated on the T1–T7 region of the spine. (India)

COVID-19 = coronavirus disease of 2019; EU = European Union; LMI = lower-middle income; TST = tuberculin skin test; UMI = upper-middle income.

## DISCUSSION

In addition to posing a possibly life-threatening risk, TB can place severe stresses on affected individuals in everyday life through symptoms, emotional and social impacts, activity limitations, the difficulties associated with treatment, and potentially long-term sequelae. Studies have explored the experience of TB in single geographic locations, many using interview and focus group methodology in relatively small study populations (n < 50 participants).^18–30^ The focus of such research is typically understanding barriers to medical care for individuals with TB in the local context. Our research was designed to capture published data and reports from people with TB and caregivers across a wide spectrum of geographies, cultures/languages, and economic development levels, with the goal of developing a conceptual model of the personal experience of TB that can be applied to different global populations.

We found that TB is associated with a variety of symptoms and life impacts (e.g., physical, social/interpersonal, and psychological) across global populations. However, there are differences by national income level in how psychological and social impacts are experienced. For example, mentions of anxiety and suicidal ideation were found in the literature review for lower-middle, upper-middle, and high-income nations but not low-income nations. Cultural factors also may shape the experience of TB management, with some affected individuals fearing that diagnosis will result in stigmatisation or expressing hopelessness about treatment efficacy. Misconceptions about TB transmission were more frequent at lower national income levels.

People with TB and caregivers across all income levels reported delays in TB diagnosis and treatment. They also frequently raised adverse effects of treatment as a concern. For those from lower-income nations, financial and other obstacles to medical access were the chief concern.

The findings of the linguistic analysis of social media were largely consistent with those of the literature review. A few additional symptoms were mentioned in social media, and the impact of the COVID-19 pandemic was a new finding. Across national income levels, people with TB posted about the similarity of TB and COVID-19 symptoms as an obstacle to diagnosis and treatment.

There were limitations to our approach. The measurement and interpretation of concepts is limited by potential biases based on culture, socio-economics, and language selectivity (predominantly English). For example, although our research did not find mentions of anxiety and suicidal ideation in low-income countries, this may have been because of a lesser chance of being assessed for psychological comorbidities. Online sources may be prone to self-selection bias, given that people are often more likely to report negative experiences. Relatively few of the published articles and patient stories/blogs originated in low-income countries. The representativeness of the social media linguistic analysis is constrained by the digital access and literacy of people with TB and caregivers, and the conclusions rely on unverified diagnosis and treatment status. Further, the literature review data are relatively dated, and unlike the social media analysis, did not account for contemporary challenges posed by COVID-19.

## CONCLUSION

The conceptual model developed here can inform future research on the impacts of TB and its treatment on the individual. The results indicate the broad array of symptoms that must be considered and the multiplicity of the life dimensions affected, as well as how geography, culture, and income or socio-economic status can shape the experience of people with TB. Lack of specific and globally accepted indicators is a challenge for monitoring people’s experience and hence responding to their needs and addressing challenges. International guidance documents with input from TB communities are needed. Guidelines on the development of patient-reported outcomes assessment instruments indicate the importance of identifying concepts important to affected individuals early in the development process.^13^ Within this context, our data will enable the development of assessment instruments appropriate to capture the personal experience of TB in clinical trials that span different world populations.

## Supplementary Material





## References

[1] World Health Organization. Global tuberculosis report 2024. Geneva: WHO, 2024.

[2] Yasobant S, Health-related quality of life (HRQoL) of patients with tuberculosis: a review. Infect Dis Rep. 2022;14(4):509-524. 35893474 10.3390/idr14040055 PMC9326555

[3] Alsayed SSR, Gunosewoyo H. Tuberculosis: pathogenesis, current treatment regimens and new drug targets. Int J Mol Sci. 2023;24(6):5202. 36982277 10.3390/ijms24065202 PMC10049048

[4] Alene KA, Interventions to prevent post-tuberculosis sequelae: a systematic review and meta-analysis. EClinicalMedicine. 2024;70:102511. 10.1016/j.eclinm.2024.102511 PMC10907188 38434448

[5] Wong YJ, Impact of latent tuberculosis infection on health and wellbeing: a systematic review and meta-analysis. Eur Respir Rev. 2021;30(159):200260. 10.1183/16000617.0260-2020 PMC9489106 33408089

[6] Fox GJ, Preventive therapy for latent tuberculosis infection-the promise and the challenges. Int J Infect Dis. 2017;56:68-76. 10.1016/j.ijid.2016.11.006 27872018

[7] Shedrawy J, Quality of life of patients on treatment for latent tuberculosis infection: a mixed-method study in Stockholm, Sweden. Health Qual Life Outcomes. 2019;17(1):158. 10.1186/s12955-019-1228-4 PMC6813984 31651339

[8] United Nations General Assembly. Seventy-third session, agenda item 129. New York, NY, USA: United Nations Digital Library, 2018. https://digitallibrary.un.org/record/1645268?ln=en.

[9] Shete PB, Reid M, Goosby E. Message to world leaders: we cannot end tuberculosis without addressing the social and economic burden of the disease. Lancet Glob Health. 2018;6(12):e1272-e1273. 10.1016/S2214-109X(18)30378-4 30224288

[10] Addo J, Living with tuberculosis: a qualitative study of patients’ experiences with disease and treatment. BMC Public Health. 2022;22(1):1717. 36085073 10.1186/s12889-022-14115-7 PMC9462890

[11] Hoos A, Partnering with patients in the development and lifecycle of medicines: a call for action. Ther Innov Regul Sci. 2015;49(6):929-939. 26539338 10.1177/2168479015580384 PMC4616907

[12] Mercieca-Bebber R, The importance of patient-reported outcomes in clinical trials and strategies for future optimization. Patient Relat Outcome Meas. 2018;9:353-367. 30464666 10.2147/PROM.S156279 PMC6219423

[13] Patrick DL, Content validity–establishing and reporting the evidence in newly developed patient-reported outcomes (PRO) instruments for medical product evaluation: ISPOR PRO good research practices task force report: part 1–eliciting concepts for a new PRO instrument. Value Health. 2011;14(8):967-977. 22152165 10.1016/j.jval.2011.06.014

[14] U S Department of Health and Human Services FDA Center for Drug Evaluation and Research; U.S. Department of Health and Human Services FDA Center for Biologics Evaluation and Research; U.S. Department of Health and Human Services FDA Center for Devices and Radiological Health. Guidance for industry: patient-reported outcome measures: use in medical product development to support labeling claims: draft guidance. Health Qual Life Outcomes. 2006;4:79. 17034633 10.1186/1477-7525-4-79 PMC1629006

[15] European Medicines Agency. Appendix 2 to the guideline on the evaluation of anticancer medicinal products in man -The use of patient-reported outcome (PRO) measures in oncology studies – scientific guideline. Amsterdam, The Netherlands: European Medicines Agency, 2016. https://www.ema.europa.eu/en/appendix-2-guideline-evaluation-anticancer-medicinal-products-man-use-patient-reported-outcome-pro-measures-oncology-studies-scientific-guideline.

[16] International Council for Harmonization of Technical Requirements for Pharmaceuticals for Human Use (ICH). ICH harmonized guidance general considerations for clinical studies E8(R1). Geneva, Switzerland: ICH, 2021. https://database.ich.org/sites/default/files/E8-R1_Guideline_Step4_2021_1006.pdf.

[17] U.S. Department of Health and Human Services. FDA patient-focused drug development guidance series for enhancing the incorporation of the patient’s voice in medical product development and regulatory decision making. Silver Spring, MD, USA: Center for Drug Evaluation and Research, 2024.

[18] Anthoney J, Patients’ perspectives on factors facilitating adherence to tuberculosis treatment in Iquitos, Peru: a qualitative study. BMC Health Serv Res. 2021;21(1):345. 33853587 10.1186/s12913-021-06329-z PMC8048224

[19] Baral SC, The importance of providing counselling and financial support to patients receiving treatment for multi-drug resistant TB: mixed method qualitative and pilot intervention studies. BMC Public Health. 2014;14:46. 24438351 10.1186/1471-2458-14-46 PMC3898066

[20] Bhattacharya Chakravarty A, Such a long journey: what health seeking pathways of patients with drug resistant tuberculosis in Mumbai tell us. PLoS One. 2019;14(1):e0209924. 30653523 10.1371/journal.pone.0209924 PMC6336307

[21] Bieh KL, Weigel R, Smith H. Hospitalized care for MDR-TB in Port Harcourt, Nigeria: a qualitative study. BMC Infect Dis. 2017;17(1):50. 28068907 10.1186/s12879-016-2114-x PMC5223486

[22] Choowong J, Tillgren P, Söderbäck M. Thai people living with tuberculosis and how they adhere to treatment: a grounded theory study. Nurs Health Sci. 2017;19(4):436-443. 28719050 10.1111/nhs.12362

[23] Cremers AL, Tuberculosis patients’ pre-hospital delay and non-compliance with a longstanding DOT programme: a mixed methods study in urban Zambia. BMC Public Health. 2016;16(1):1130. 27793145 10.1186/s12889-016-3771-9 PMC5086075

[24] Daftary A, Padayatchi N, O’Donnell M. Preferential adherence to antiretroviral therapy over tuberculosis treatment: a qualitative study of drug-resistant TB/HIV co-infected patients in South Africa. Glob Public Health. 2014;9:1107-1116. 10.1080/17441692.2014.934266 PMC4198425 25035943

[25] de Andrade ET, Perspectives of patients, doctors and medical students at a public university hospital in Rio de Janeiro regarding tuberculosis and therapeutic adherence. PLoS One. 2015;10(9):e0137572. 26360291 10.1371/journal.pone.0137572 PMC4567070

[26] Gebreweld FH, Factors influencing adherence to tuberculosis treatment in Asmara, Eritrea: a qualitative study. J Health Popul Nutr. 2018;37:1. 10.1186/s41043-017-0132-y PMC5756387 29304840

[27] Li SP, Barriers to tuberculosis care for drug users in two provinces of China: a qualitative study. Int J Tuberc Lung Dis. 2013;17(10):1358-1363. 24025390 10.5588/ijtld.12.0784

[28] Shatil T, What constitutes health care seeking pathway of TB patients: a qualitative study in rural Bangladesh. J Epidemiol Glob Health. 2019;9(4):300-308. 31854173 10.2991/jegh.k.190929.001 PMC7310790

[29] Tupasi T, Multidrug-resistant tuberculosis patients’ views of interventions to reduce treatment loss to follow-up. Int J Tuberc Lung Dis. 2017;21(1):23-31. 28157461 10.5588/ijtld.16.0433 PMC5427639

[30] Zuñiga JA, Mexican American men’s experience of living with tuberculosis on the U.S.-Mexico border. Am J Mens Health. 2016;10(1):32-38. 10.1177/1557988314555359 25359869

