# Supplementary material for: Conceptualising the experience of having TB: a global qualitative study
Source: IJTLD Open. 2026 Jan 9;3(1):38–45. doi: 10.5588/ijtldopen.25.0112 (PMC12810751; doi:10.5588/ijtldopen.25.0112)
Supplement: Supplementary file 1 [file ijtldopen25-0112_supplementarydata1.pdf]

Supplemental Material

Supplemental Methods

Search for web-based stories/blogs

A targeted review of websites identified via search engine was conducted. Websites reporting on the patient experience of living with tuberculosis (TB) were reviewed to identify patient blogs/stories, available in English, written by individuals with all types of pulmonary TB. Websites known to the research team were also pre-identified and included in the review (Doctors without Borders, TB Alert, TB Alliance, TB Europe Coalition, US Centers for Disease Control and Prevention). Blogs/stories were deemed eligible if they detailed concepts of interest reported from the patient perspective including information on the signs, symptoms, treatment experiences, and impacts of the disease. Although blogs were reviewed in the English language, the selected websites chosen also included blogs in other languages that had been translated into the English language, in order to allow for capture the experience of TB across cultures. It is noted that the following websites were not included in the review as they did not contain patient-reported blogs specific to TB: American Thoracic Society (ATS), Each Breath (a blog by the American Lung Association), Treatment Action Group, and TBpeople.

Search for social media posts

A third-party data aggregator was used to identify relevant social media posts. A Boolean search string was leveraged to search for relevant posts and included English, German, Spanish, French, Italian, and Portuguese versions of the condition name, "tuberculosis." The posts were manually coded by analysts fluent in the language of the post; hence, no tool or technology was used for translation or coding.

Eligibility criteria for the posts were:

1) Discussed a specific personal experience with one of the following: TB symptoms, impact of having TB, treatment burden of TB, and/or coping mechanisms for having TB. Post authors could be people with TB, caregivers, or clinicians
2) Posted from January 1, 2020, through November 30, 2022
3) Posted in English, German, Spanish, French, Italian, or Portuguese
4) Made in one of the following countries: Brazil, France, Germany, India, Indonesia, Italy, Nigeria, Pakistan, Peru, Philippines, South Africa, Spain, Thailand, United Kingdom, United States

The location of a post's author was determined by the location included in the metadata of the post, self-described residence in the bio of the posting author, or self-described residence within a post as made by the author.

Conceptual model development

Development of the conceptual model of the personal experience of having TB was an iterative process. An initial coding framework to capture symptoms, impact, and treatment experiences was generated in an exploratory phase in which the captured data was reviewed and the categories for each section of the framework were defined. From there, the framework was validated by another researcher who coded the data and compared results with the researcher who developed the framework. Upon alignment, a detailed training was developed for additional researchers to ensure consistent coding. The social media analysis followed the same process for coding of content, with an additional consideration for sources being in multiple languages, which required that each post was manually reviewed and coded by a researcher fluent in the language in which the post was made.

Figure S1. Selection process for (A) published articles and (B) web-based patient stories and blogs

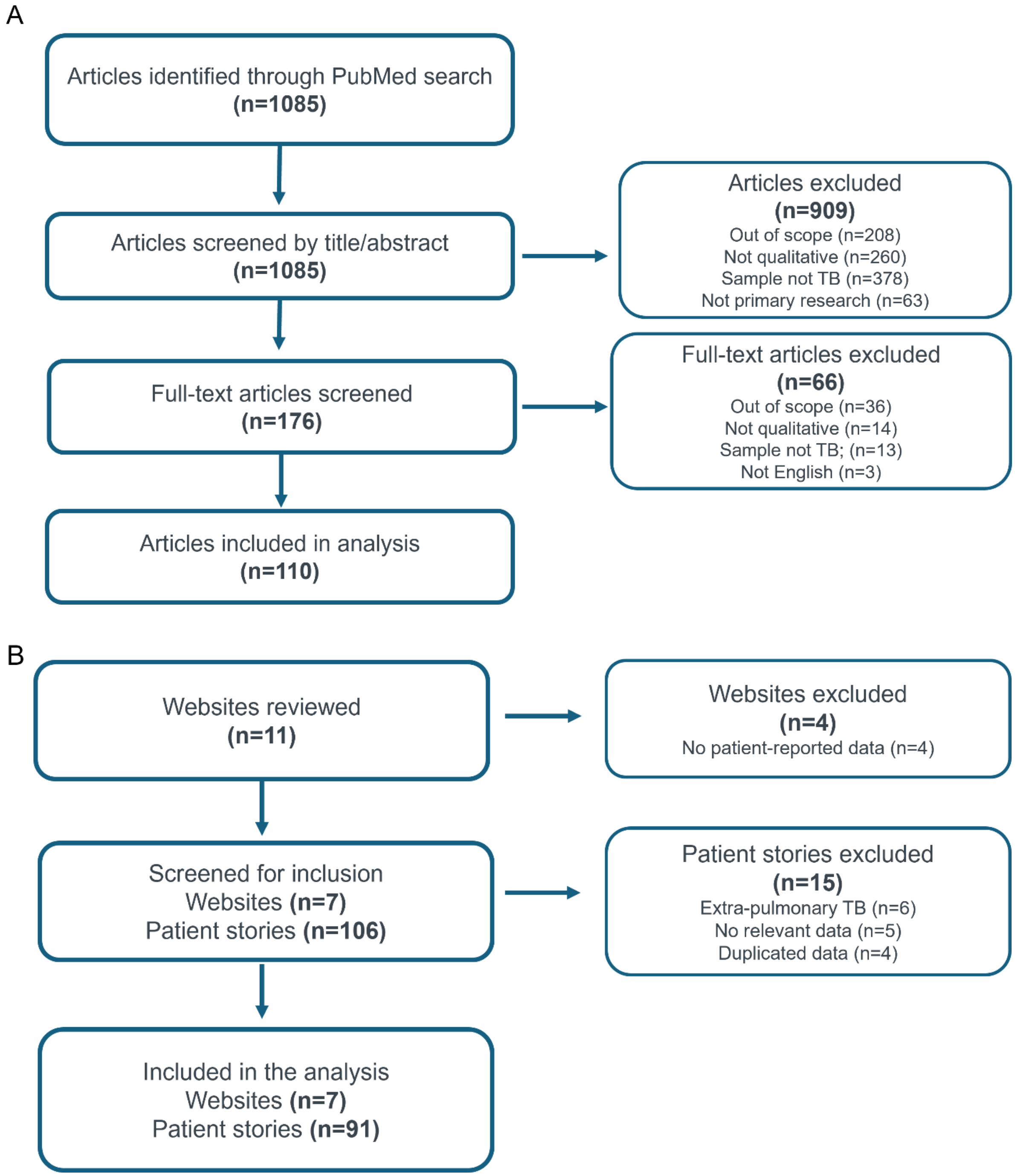

Figure S2. Source countries for published articles (n=110) and web-based patient stories/blogs (n=91) included in the literature review

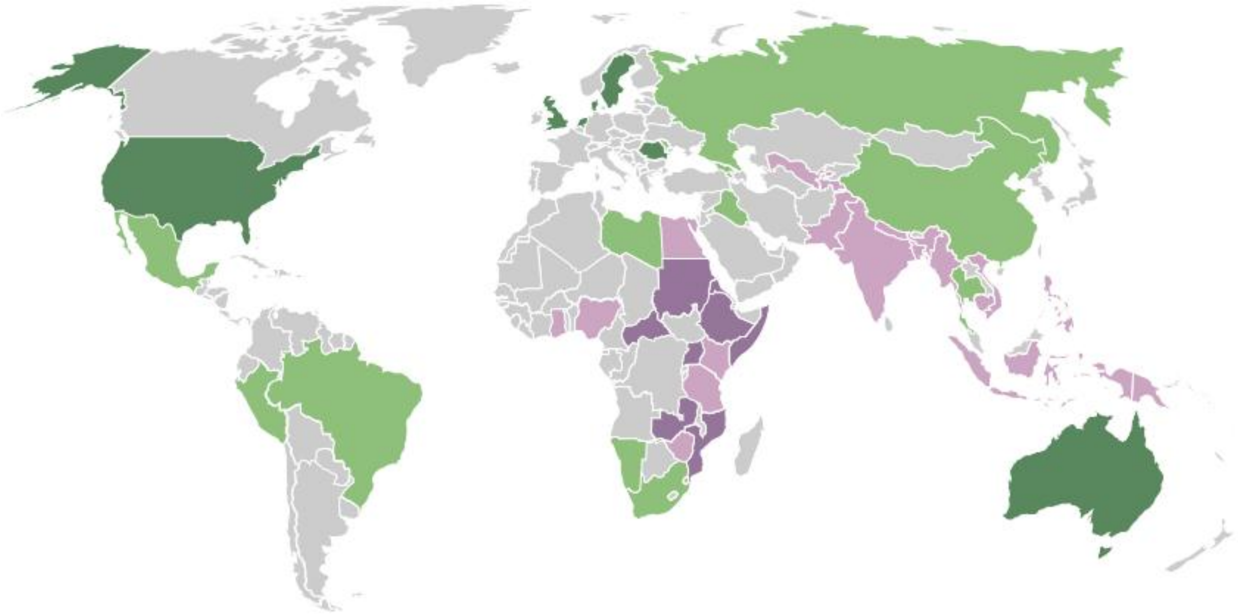

Per Capita Income Level[a] (US Dollars)	Low (<$1,085)	Lower – middle ($1,086 – $4,255)	Upper – middle ($4,256 – $13,205)	High (>$13,205)
	Ethiopia	India	South Africa	United States
	Somalia	Myanmar	Brazil	United Kingdom
	Zambia	Eswatini	China	Australia
	Uganda	Nepal	Iraq	Denmark
	Central African Republic	Bangladesh	Russia	Netherlands
	Eritrea	Kenya	Peru	Romania
	Mozambique	Nigeria	Armenia	Sweden
	Sudan	Pakistan	Mexico	
		Papua New Guinea	Namibia	
		Philippines	Thailand	
		Tajikistan	Georgia	
		Tanzania	Libya	
		Uzbekistan	Moldova	
		Zimbabwe		
		Cambodia		
		Egypt		
		Ghana		
		Indonesia		
		Vanuatu		
		Vietnam		

[a]World Bank 2022-2023 income classification based on gross national income per capita of the previous year.

Figure S3. Symptoms and impacts of TB by national income level in social media posts

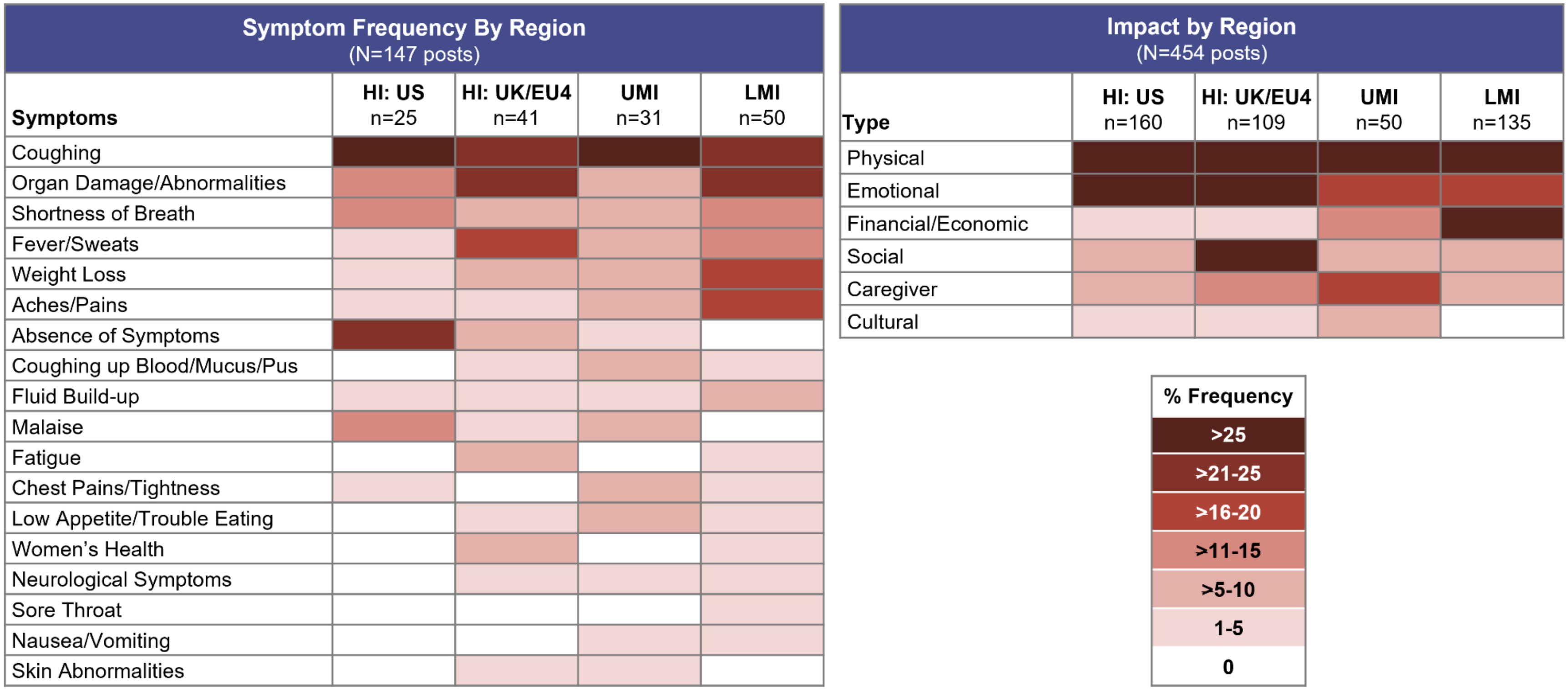

HI, high income (United States, European Union/United Kingdom [France, Italy, Spain, Germany, United Kingdom]); LMI, lower-middle income (India, Bangladesh, Nigeria, Pakistan, Philippines, Cambodia, Indonesia); TB, tuberculosis; UMI, upper-middle income (South Africa, Brazil, Peru, Thailand).

Figure S4. Experience of TB diagnosis and treatment by national income level in social media posts

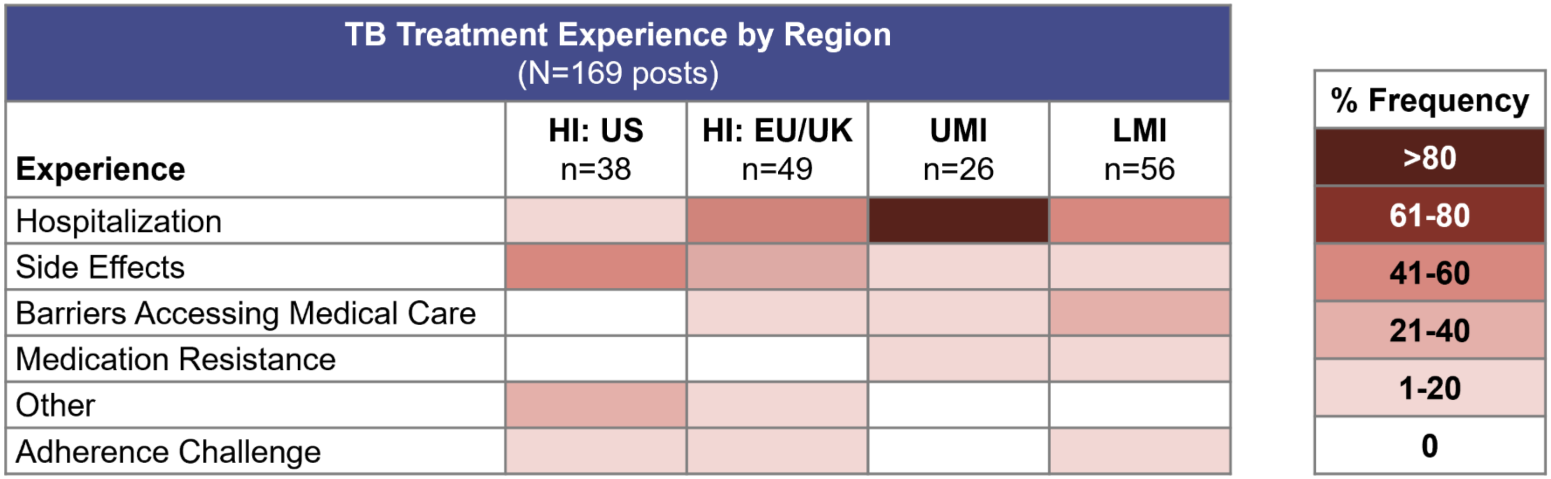

EU/UK, European Union/United Kingdom (France, Italy, Spain, Germany, United Kingdom); HI, high income; UMI, upper-middle income; LMI, lower-middle income; TB, tuberculosis.
